# Elastic Shape Memory Hybrids Programmable at Around Body-Temperature for Comfort Fitting

**DOI:** 10.3390/polym9120674

**Published:** 2017-12-04

**Authors:** Tao Xi Wang, Chris Renata, Hong Mei Chen, Wei Min Huang

**Affiliations:** 1 School of Mechanical and Aerospace Engineering, Nanyang Technological University, 50 Nanyang Avenue, Singapore 639798, Singapore; WA0003XI@e.ntu.edu.sg (T.X.W.); rchristianto@ntu.edu.sg (C.R.); 2 College of Chemistry and Materials Science, Sichuan Normal University, Chengdu 610066, China; chenhongmei@sicnu.edu.cn

**Keywords:** shape memory hybrid, elasticity, comfort fitting, hardening time, shape memory effect

## Abstract

A series of silicone based elastic shape memory hybrids are fabricated. Their shape memory performance, mechanical behaviors at room temperature with/without programming and during fitting at 37 °C are investigated. It is found that these materials have good shape memory effect and are always highly elastic. At 37 °C, there are 10 min or more for fitting. Thus, it is concluded that this type of material has great potential as an elastic shape memory material for comfort fitting.

## 1. Introduction

Nowadays, people are willing to invest more for their own personal fitness. Comfort fitting becomes a more and more important consideration in choosing personal products, such as clothing, footwear, insoles and orthotic devices. Take the footwear market as an example, customers start to search for footwear with better comfort and more functionality [1]. Thus, in the past decade many customized shoes have emerged that are targeted towards different sports activities, such as basketball, football and skiing. People trust more on such customized footwear, which are designed to provide better protection and prevent injury due to mis-fitting resulted by undue pressure on the foot from tightly fitting footwear or unwanted friction from loosely fitting footwear [2,3,4].

On the other hand, purposely designed orthotic devices have been applied successfully for treatment of lower extremity injuries [5]. From the medical point of view, personalized or semi-personalized products can offer better rehabilitative effect for individuals [6]. Although such personalized devices are preferred, their high cost is a big burden to many patients.

Although right now some new technologies have been developed to customize footwear or other wearable items for individuals, almost all of them have some apparent weakness either within the product itself or in the manufacturing process. For instance, although 3D printing technology is claimed to be able to provide products which perfectly follow the general contour of human body [7,8], the tedious process of 3D scanning and the high cost in printing largely limit its possibility to move forward at this moment. The same scenario occurs in the industry of orthotic devices as well, as the cost for customization has yet been reduced to the level, which is affordable for everyone.

Therefore, a cost-effective and easy-realizable approach to achieve better comfort fitting, not only limited to the applications mentioned above but also for a wider range of wearable personal products, is highly in demand at present. 

The so called shape memory materials (SMMs) may provide the just right solution for such comfort fitting applications. Basically, SMMs are featured by their unique ability to return their original shape only when the right stimulus, such as heat (thermo-responsive), chemical (chemical-responsive), light (photo-responsive), is applied [9,10,11,12,13,14,15]. This interesting phenomenon is known as the shape memory effect (SME). Traditionally, shape memory alloy (SMA) and shape memory polymer (SMP) are two most well-known types of SMMs and they have been widely used for a number of engineering applications, such as medical engineering [16,17,18,19,20], surface patterning [21,22,23,24,25] and aerospace engineering [26,27].

In fact, shape memory insoles made of shape memory polymeric foams are already commercially available in the market [28]. According to [29], utilizing the heat-responsive SME, shape memory insole is able to self-adjust its shape to meet the needs of different foot pressure for individuals. However, heating to higher temperatures, which are well above our body temperature, is required during fitting, which unavoidably involves two potential problems, namely over-heating and limited time for fitting within the right temperature window during cooling [30].

Apparently, in addition to the heating-responsive SME and good elasticity during and after fitting as in current shape memory insoles, an ideal SMM for enhanced comfort fitting experience must have the additional feature of prolonged time period for fixing the temporary shape during fitting (technically called programming within the community of SMMs) at around body temperature.

In recent years, the concept of shape memory hybrid (SMH) has been proposed to add in a new dimension in developing new SMMs to meet the needs of particular engineering applications [31]. A SMH normally includes at least two components, one is elastic component and the other is the transition component. As explained in [32], the elastic component is always highly elastic and stores the elastic energy after programming, while the transition component is able to soften upon heating to above the transition temperature and then largely maintains the deformed shape after cooling for hardening. Upon heating to soften the transition component again, the elastic energy stored in the elastic component is released, which provides the drive force for shape recovery of the hybrid. The particular feature of SMHs is that these two components are carefully selected to avoid any interaction between them.

By carefully selecting those two components and the process to mix them together without inducing any chemical interaction between them (or with minimized interaction), the mechanical properties and commonly observed shape memory features in most of SMAs and SMPs (such as dual-SME, triple-SME, two-way reversible SME, thermo-responsive, moisture- responsive) of the resulted hybrid can be pre-determined [33].

In addition, due to the flexibility in selecting the components for SMHs, some special features, such as water-responsive [34], cooling-responsive [35] and healing [36], become a matter of conventional. Furthermore, if highly elastic silicone is utilized as the elastic component, rubber-like SMHs at both high and low temperatures have been realized in [37]. However, because the transition component in [37] is made of melting glue, in which the melting and crystallization temperatures are 70 °C and 55 °C, respectively, the programming temperature to fix the temporary shape (e.g., for fitting) is well above 37 °C and the time window for programming is also too short for comfit fitting applications.

In this paper, based on the concept of SMH, a new type of SMH, namely SPPM, is developed and characterized in terms of the heating-responsive SME in particular for features mentioned above in comfort fitting applications.

## 2. Material, Sample Preparation and Experimental

Same as that used in the elastic SMH reported in [36,37], polydimethylsiloxane (PDMS or SYLGARD^®^ 184) from Dow Corning Corporation (Midland, MI, USA) was used as the elastic component. It is highly compatible with many materials and can well maintain its excellent elasticity within a wide temperature range from −50 to 200 °C. 

The SYLGARD^®^ 184 silicone elastomer kit comes with two parts, namely the base material (resin monomer) and curing agent. Both parts are in liquid form. A volume ratio of 10 (base material) to 1 (curing agent) is recommended by the manufacturer for mixing and then curing at room temperature or high temperatures.

However, instead of using melting glue as in [36,37], five different transition components were used in the course of this study. According to the datasheet provided by the supplier, the major compositions of the so called PPM series in pellet form from Nanjing Licong New Materials Ltd. (Nanjing, Jiangsu, China) are polyurethane (PU) and ethylene-vinyl acetate (EVA). Refer to Table 1 for the wt % of PU in each PMM model. It was found that PPMs are very similar to poly-ε-caprolactone (PCL) and can be crystallized at human body temperature.

**Figure 1:**
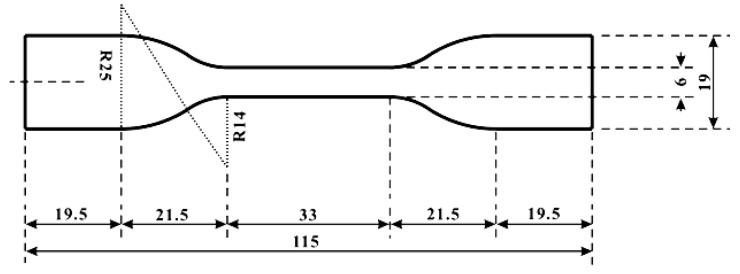
Dimensions of the sample (unit: mm). The thickness of the sample is 6 mm. Five different types of samples/hybrids—named SPPM*x* (*x* = 1 to 5)—were produced. Refer to Table 1 for their actual compositions.

In the fabrication process, a particular PPM and base material of the silicone elastomer were weighted according to the specified weight ratio and then put into a beaker together. Subsequently, the mixture was heated to 130 °C in an oven. Stirring at a high speed for five minutes was necessary to achieve good dispersion of PPM. To prevent PPM from aggregating together, it was a must to keep on stirring during cooling until PPM becomes solid. After the mixture was cooled down to room temperature (about 23 °C), curing agent was added in and mixed thoroughly again at room temperature. During mixing, many air bubbles might be generated and moisture from air might be trapped inside. Hence, a vacuum de-airing process was applied by means of placing the mold in a vacuum chamber with a pressure of 8 mbar at room temperature for ten minutes and then in air for another two minutes. Due to high viscosity nature of the mixture, this process should be repeated several times to ensure perfect mixing. In the last step, the mixture was poured into a polytetrafluoroethylene (PTFE) mold with the specified dimensions to produce dumbbell-shaped samples—as shown in Figure 1—following the ASTM D638 standard (type IV) for uni-axial tensile test or other sized molds required by the particular tests and then the mold was kept in air at room temperature for 24 h for curing.

**Figure 2:**
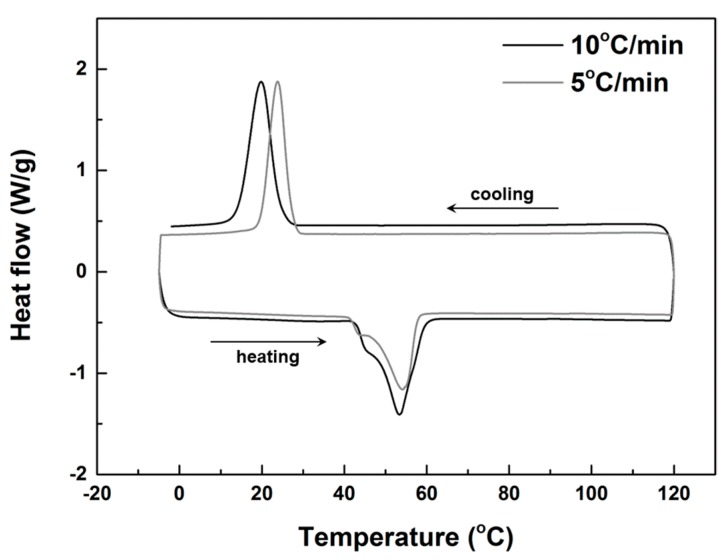
DSC results of PPM002040 at heating/cooling speeds of 10 °C/min (black line) and 5 °C/min (grey line).

Since the two components of a SMH are selected to avoid any interaction, the transition temperatures of a SPPM should be the same as those of the used PPM. Figure 2 is the differential scanning calorimetry (DSC) result of PPM002040 upon thermal cycling between −5 and 120 °C at two speeds of 5 °C/min (grey line) and 10 °C/min (black line), respectively, using a Q200 DSC machine from TA Instruments (New Castle, DE, USA). As we can see clearly, two peaks are observed in each curve representing melting upon heating and crystallization upon cooling, respectively. Thus, the melting temperature range may be defined as from 40 and 65 °C. However, the peak temperatures for crystallization differ by about 5 °C in these two tests, which reveals that the typical testing speed effect observed in many materials in DSC test is more applicable only in the cooling process of this PPM.

DSC results of other PPMs are similar to PPM002040. In fact, as we have observed, given enough time (e.g., 30 min), all PPMs were able to crystallize at 37 °C (our body temperature). 

**Figure 3:**
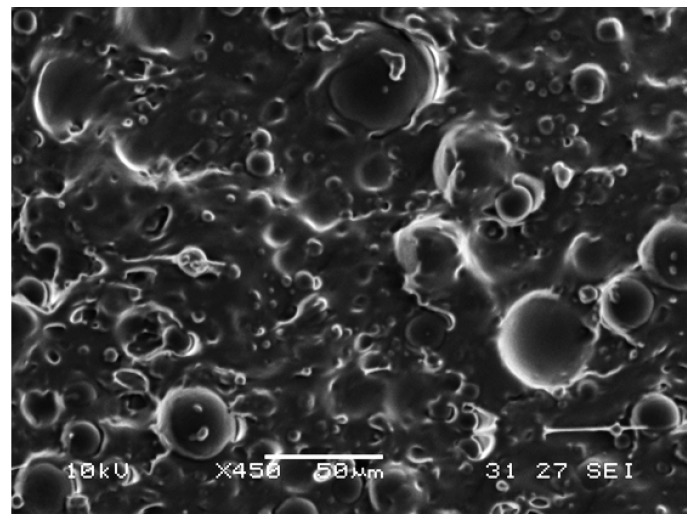
Typical scanning electron microscope (SEM) image of SPPM4. The scale bar is 50 µm.

Figure 3 is the scanning electron microscope (SEM) image of SPPM4 captured by a JOEL 7600F field emission scanning electron microscope (FESEM) from JEOL, Ltd. (Tokyo, Japan). As we can see, similar to that reported in [37], the spherical balls with a diameter from about 40 µm to about 5 µm are PPM inclusions, which are dispersed rather evenly with apparent aggregation within the silicone matrix.

In order to characterize a SMM for comfort fitting applications, it is necessary to test not only its shape memory responsive but also its mechanical behavior during and after programming.

An Instron 5565 testing machine from Instron Engineering Corporation (Norwood, MA, USA) (with a thermal chamber for temperature control) was used to stretch room temperature SPPM samples till fracture at a strain rate of 10^−2^/s to find their maximum capability in uni-axial tension. 

Note that unless otherwise stated, herein, the stress and strain used in this study are meant for engineering stress and engineering strain.

Subsequently, a series of uni-axial cyclic tensile tests (to 10%, 30% or 60% strain in an ascending order with 3 cycles for each strain) were carried out to investigate the elastic response of SPPM samples at room temperature. The applied strain rate in both loading and unloading was 10^−2^/s.

The shape memory performance of SPPM samples was investigated by means of the standard thermo-responsive SME cycle, which includes two processes (namely, programming and recovery) in three steps [38]. Note that as we have observed, relaxation/creeping is not significant in these materials.

In the first step, a sample was pre-heated to 80 °C to be fully softened. Only when the sample was cooled to 37 °C (body temperature), uni-axial tension was started until a prescribed maximum programming strain (*ε_m_*) was reached. The thermal chamber was used to maintain the sample at 37 °C. In the second step, unloading was carried out 30 min later after the sample was fully crystallized. The residual strain was denoted by *ε*_1_. This ended the programming process. In the last step, the sample was heated to 50 °C in an oven for five minutes. The remaining strain was denoted by *ε*_2_. This was the end of the recovery process.

Two maximum programming strains of 20% and 50% were applied in this study and the loading/unloading strain rate was 10^−3^/s.

A set of cyclic tests were conducted to estimate the time required for full crystallization at 37 °C. Three cycles (all to 20% strain with a holding period of eight minutes in-between) were carried out at a strain rate of 10^−1^/min in each test. 

The elasticity of the SPPM samples after programming was also examined. Cyclic uni-axial tensile tests were carried out to 10% strain in the first cycle and then 30% strain in the second cycle at room temperature on the programmed samples with two different programmed strains of 20% and 50%. The applied strain rate was 10^−2^/s.

## 3. Experimental Results and Analysis

Most of the tests were repeated three times. Herein, we only show typical ones, while all testing results were used in analysis whenever applicable.

**Figure 4:**
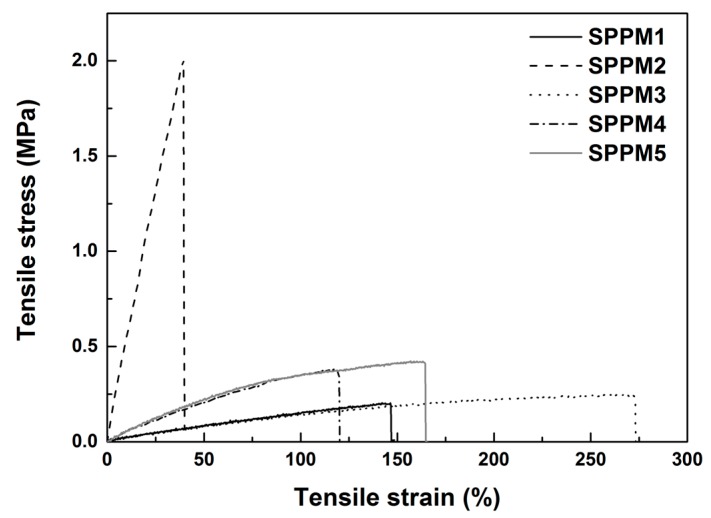
Typical stress vs. strain relationships of SPPM samples under uni-axial tension at room temperature to fracture.

Figure 4 presents typical stress vs. strain relationships of all samples in uni-axial tension till fracture. As we can see, SPPM2 is outstanding as it is much harder than all the others and its fracture strain is much less, less than 50%. All other four samples are able to stretch by over 100% without fracture. As the required stress (well below 0.5 MPa) is low, we may claim that all samples except SPPM2 are soft and highly stretchable. 

**Figure 5:**
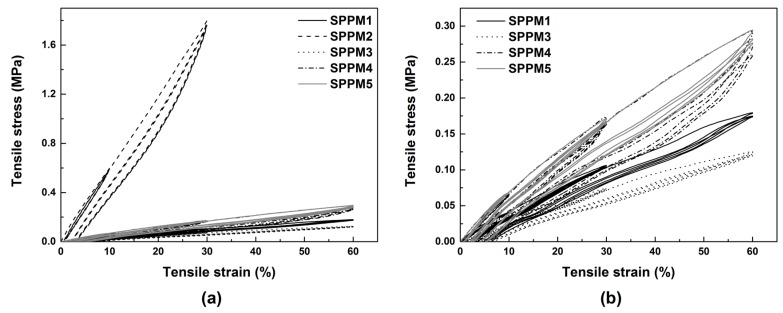
Typical stress vs. strain relationships under uni-axial cyclic tension at room temperature of all samples (**a**) and zoom-in view of all samples excluding SPPM2 (**b**).

Typical stress vs. strain relationships of all samples in cyclic tension at room temperature are plotted together in Figure 5 for comparison. Since the fracture strain of SPPM2 is less than 50% (as revealed in Figure 4), the cyclic strains applied on SPPM2 are 10% and 30%. As we can see, all SPPMs are highly elastic upon cyclic stretching at room temperature with very small residual strain. A close look of Figure 5 reveals that the residual strain in the first cycle upon reaching the prescribed strain is larger than that in the subsequent cycles to the same strain. This is due to the de-bonding effect in the first cycle as reported in [37].

**Figure 6:**
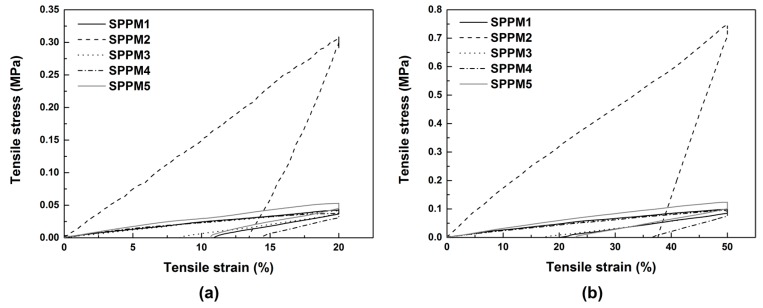
Typical stress vs. strain relationships of all samples programmed with 20% strain (**a**) and 50% strain (**b**).

**Figure 7:**
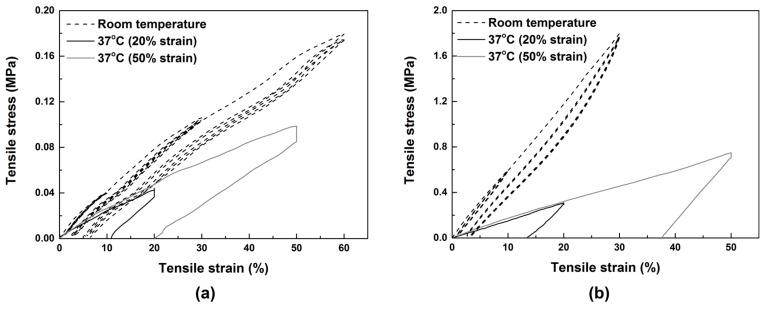

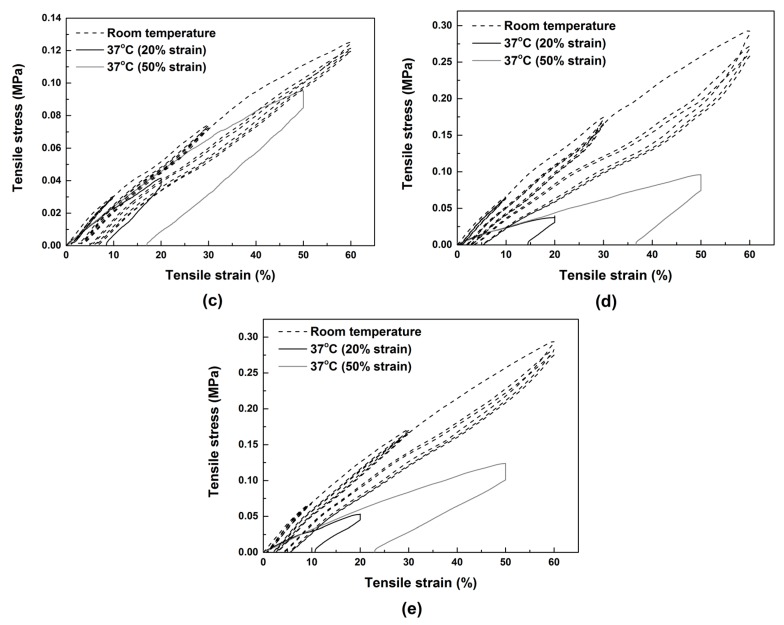
Comparison of the stress vs. strain relationships under uni-axial tension at room temperature and 37 °C (to 20% and 50% strains) in SPPM1 (**a**), SPPM2 (**b**), SPPM3 (**c**), SPPM4 (**d**) and SPPM5 (**e**).

The stress vs. strain relationships of all samples during programming are presented in Figure 6. In Figure 7, we compare them individually with their respective stress vs. strain relationships at room temperature as shown in Figure 5.

**Figure 8:**
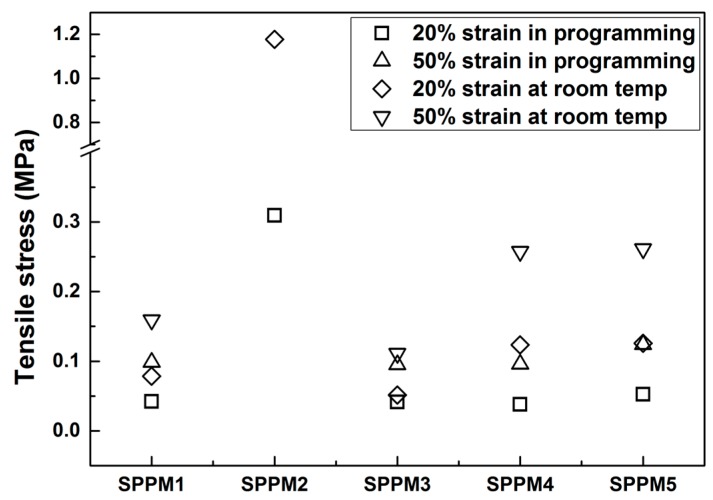
Comparison of the corresponding stresses at 20% and 50% strains in loading at room temperature and in programming (at 37 °C).

Based on the experimental results reported in Figure 6 and Figure 7, the stresses corresponding to 20% and 50% strains in loading are compared in Figure 8. Note that for SPPM2, the maximum strain in stretching at room temperature is only 30% strain (refer to Figure 7b), so that the results of 50% strain of this sample is not included. It can be seen that while in SPPM3, the difference is almost negligible, so that the material is always very soft, the other materials become significantly harder, in particular for SPPM2, in which after hardening, the stress increases by three times from 0.3 to 1.2 MPa at 20% strain. For SPPM4 and SPPM5, the stresses at 20% and 50% are all about doubled after programming.

**Figure 9:**
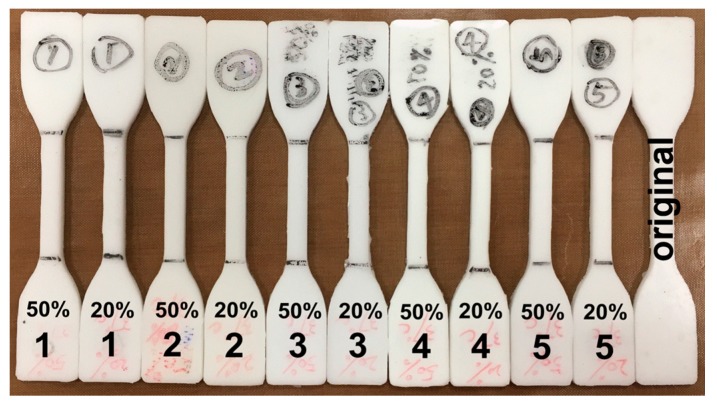
SPPM samples after heated at 50 °C for five minutes. In each sample, the applied programming strain is indicated at right above the hybrid type (1 to 5). The right sample is original as marked without any testing for comparison.

After programming, all programmed samples were heated at 50 °C for five minutes. Subsequently all samples were aligned together as shown in Figure 9. When compared with the original sample without testing (the right piece in Figure 9), almost full shape recovery is observed in all tested samples.

The shape fixity ratio (*R_f_*) and shape recovery ratio (*R_r_*), which are defined by,

(1) $$R_{f}=\frac{\varepsilon_{1}}{\varepsilon_{m}}$$

(2) $$R_{r}=\frac{\varepsilon_{1}-\varepsilon_{2}}{\varepsilon_{1}}$$

can be applied to qualitatively evaluate the shape memory performance of SMMs [38].

**Figure 10:**
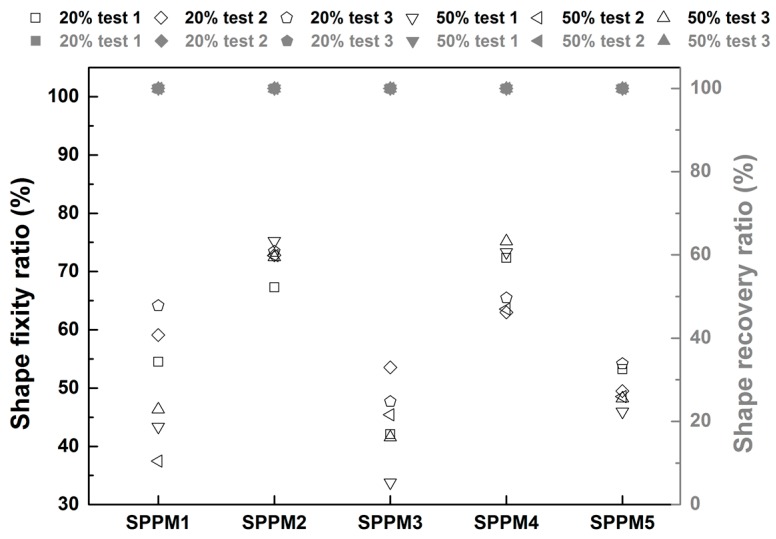
Shape fixity ratio and shape recovery ratio of all SPPM samples (black hollow symbols: shape fixity ratio; grey solid symbols: shape recovery ratio). The applied programming strain in each sample is indicated in % in the legend (top).

The shape fixity ratio and shape recovery ratio of all tests (repeated three times) are summarized in Figure 10. It is clear that while the shape recovery ratio is always 100% in all samples, the actual shape fixity ratio varies not only according to the type of the sample but also the applied programming strain. In general, both SPPM2 and SPPM4 have the highest shape fixity ratio (around 70%) for both 20% and 50% programming strains, while the rest are 50% or less. Although the stress vs. strain curves of SPPM3 during programming at 37 °C and after programming at room temperature are almost the same (refer to Figure 7c), it is still able to partially maintain its programmed shape, as the shape fixity ratio is always above 30%.

**Figure 11:**
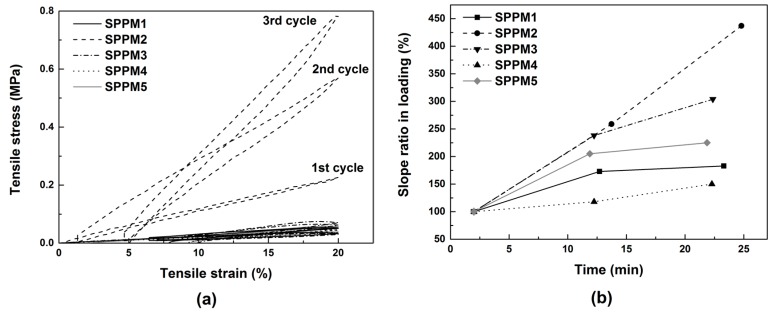
Evolution in the stress vs. strain relationship of all SPPM samples under cyclic uni-axial tension during crystallization induced hardening at 37 °C (**a**); and the slope ratios in loading at 20% strain in the loading process of each cycle as a function of testing time (**b**) (in which the slope of the stress vs. strain curve at 20% strain of the first cycle of each sample is taken as the reference).

The evolution in the mechanical response of all samples under cyclic uni-axial tension during crystallization after being cooled to 37 °C and then maintained at this temperature during testing is revealed in Figure 11a. Since SPPM2 is much stiffer than the others, the results of the other samples are difficult to trace in Figure 11a. Nevertheless, we can clearly see that SPPM2 gradually becomes stiffer during cycling. As a simple approach, it may be possible to estimate the process of crystallization induced hardening based on the slope in the stress vs. strain curve at 20% strain upon loading in each cycle. We may take the slope of the first cycle at 20% strain in loading as the reference and defined the slope ratio in loading, which is the ratio (in %) of the slope at 20% strain in a cycle to that of the first cycle.

In Figure 11b, we plot the slope ratios in loading against the testing time for all samples. While the slope ratio of SPPM 2 increases in an approximately linear manner against time—which indicates that it is still in a continuous hardening process even at least at the end of the loading process in the third cycle—the rest of the samples appear to increase moderately (e.g., in SPPM3 and SPPM4) or only slightly (e.g., in SPPM1 and SPPM5) in the last two cycles. Hence, we may claim that SPPM1 and SPPM5 are about fully crystallized from 12.5 min onwards, while SPPM3 and SPPM4 need a bit more time than half an hour to harden. But for SPPM2, the required crystallization time at 37 °C is well beyond half an hour. Since in practice, the real working temperature is normally less that 37 °C, a shorter hardening time is expected.

**Figure 12:**
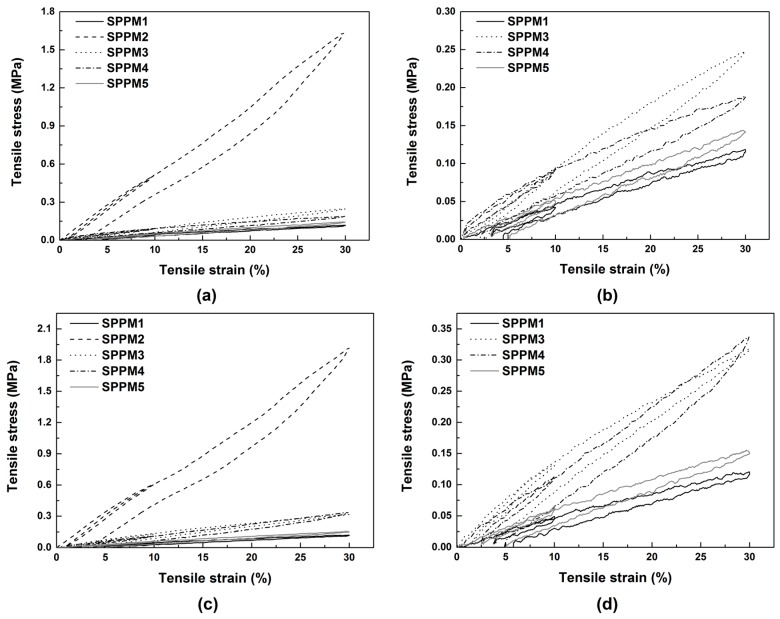
Typical stress vs. strain relationships under uni-axial cyclic tension at room temperature in all samples programmed with 20% strain (**a**) and zoom-in view for samples excluding SPPM2 (**b**); and programmed with 50% strain (**c**) and zoom-in view for samples excluding SPPM2 (**d**).

The elasticity at room temperature of the programmed samples (with two different programming strains of 20% and 50%) is revealed in Figure 12 via cyclic uni-axial tensile test to 10% maximum strain in the first cycle and then to 30% maximum strain in the second cycle. High elasticity is confirmed in all samples. Note that as mentioned above, again the residual strain observed in the samples is largely due to significant non-uniform deformation at the clamping points. It is mostly recoverable if the clamps are removed.

## 4. Conclusions

A series of elastic SMHs were fabricated. Their shape memory performance and elasticity at room temperature with/without programming were systematically investigated. In addition, the stress vs. strain relationships of these materials during fitting at 37 °C were characterized. It was found that (a) these materials are always highly elastic, not only during fitting but also after programming/fitting; (b) the elasticity of such silicone based hybrids can be adjusted if different PPMs are used; (c) the hardening time during programming at 37 °C is always more than 10 min. With relatively higher shape fixity ratios in SPPM2 and SPPM4, these two models are more capable of maintaining the deformed shape while others can provide more elastic recovery. Thus, in terms of comfort fitting, SPPM2 and SPPM4 are two most suitable and their difference in hardness is just right for different applications. Hence, we may conclude that this type of SMH has great potential as an elastic SMM for comfort fitting.

## Figures and Tables

**Table 1:** Components of five SPPM hybrids.

Sample/hybrid	wt % of silicone	Model of PPM (according to the supplier)
SPPM1	60	PPM005048 (wt % of PU: 50)
SPPM2	60	PPM000098 (wt % of PU: 0)
SPPM3	55	PPM007820 (wt % of PU: 78)
SPPM4	40	PPM003365 (wt % pf PU: 33)
SPPM5	50	PPM0060438 (wt % of PU: 60)
